# Associations between alcohol consumption and empathy in a non-clinical sample: drinking motives as a moderator

**DOI:** 10.1038/s41598-024-59233-x

**Published:** 2024-05-14

**Authors:** Isabelle C. Baltariu, Violeta Enea, Peter J. de Jong, Marije aan het Rot

**Affiliations:** 1 Department of Psychology, University of Groningen, Grote Kruisstraat 2/1, Groningen, The Netherlands; 2 Department of Psychology, Alexandru Ioan Cuza University of Iași, Iași, Romania

**Keywords:** Alcohol, Affective empathy, Cognitive empathy, Behavioral empathy, Drinking motives, Psychology, Human behaviour

## Abstract

People consume alcohol for multiple reasons. Negative motives are often associated with alcohol-related problems. These problems might be explained by negative effects of high alcohol consumption on empathy. Past studies have associated alcohol use disorder (AUD) with reduced cognitive and affective empathy. Few studies have focused on non-clinical samples and considered behavioral empathy. We examined the links between alcohol consumption and multiple aspects of empathy, and if these links were moderated by negative drinking motives. We collected online data of 520 unselected individuals. All completed the AUD Identification Test (AUDIT) and a Drinking Motives Questionnaire. Affective and cognitive empathy were assessed using the Empathy Quotient. Behavioral empathy was assessed by asking participants how likely they would help the person in each of 24 scenarios involving pain. Helping others in pain was positively predicted by affective and cognitive empathy. Higher AUDIT scores were associated with helping others less, particularly among participants who scored higher on drinking to cope with negative affect. People who drink more and do so to cope with negative affect appear to have less behavioral empathy. This supports the view that negative drinking motives contribute to AUD risk.

## Introduction

Data from the WHO^1^ indicate that over 70% of individuals aged > 15 years consume alcohol. Problematic alcohol use, defined as drinking five or more drinks on occasion for men and four or more drinks for women^2^, increases the risk for the onset of alcohol use disorder (AUD). AUD is characterized by frequent episodes of intoxication, a preoccupation with alcohol, a compulsion to consume alcohol, and a lack of control in limiting intake despite negative consequences^3^. People with AUD also have interpersonal problems, diminished social skills, and a proneness to aggressivity^4^. Interpersonal difficulties might increase the risk for spousal problems, unemployment, and homelessness. These societal issues make the study of how risky alcohol use might affect interpersonal functioning particularly relevant^5^.

The interpersonal problems of people with AUD might be explained by cognitive and emotional deficits, particularly reduced social cognition^4^. Social cognition represents one’s ability to identify, perceive, and interpret information from the social environment and comprises mental processes such as facial emotion processing, empathy, and Theory of Mind (ToM)^6^. Empathy is described by Decety and Jackson^7^ as the capacity to identify, experience, and understand the emotional states of others. Importantly, although empathy is usually evoked in real-time in response to another person's emotions or in response to socio-emotional stimuli, it may also happen retrospectively, in response to verbal statements of a third party^8^, or with stimuli involving an imaginary or fictional person^7,9–11^.

Researchers have differentiated between affective, cognitive, and behavioral forms of empathy. Affective empathy is usually defined as a person's emotional response to another person's affective state^12^. The emotional response involves feelings of compassion and concern^13^ and is often expressed instinctively^10^. In comparison, cognitive empathy is said to represent the ability to intellectually understand the feelings of others^9^ and involve the modulation of cognitive processes before expressing empathic behavior^10^. Theories on cognitive empathy underline processes such as perspective-taking and “mindreading”^14^.

Behavioral empathy is a third, less researched form of empathy. It is generally considered to be the outcome of affective and cognitive empathy processes and is often expressed as helping behavior. Behavioral empathy is thought to involve taking the perspective of another person (cognitive empathy) with subsequent helping behavior being mediated by empathic emotions, affective empathy^15^. Moreover, empathic behavior can be activated when others' negative affect (e.g., pain) leads to approaching and then helping, in order to reduce one’s own negative affect evoked by seeing another person in pain.

In line with the idea that interpersonal problems of people with AUD might be explained by deficits in social cognition, AUD has been associated with reduced facial emotion recognition and ToM^16–18^. Besides, AUD patients report having less empathy than controls^19,20^. Specifically, past findings reveal that AUD is associated with reduced affective empathy^21–23^ as well as with cognitive empathy^24–26^. Ultimately, impaired cognitive empathy in particular has been linked with risky alcohol use in both clinical^19,24,25^ and non-clinical samples^27,28^.

Besides, on self-report measures AUD patients indicate having less empathy than controls^19,20^. Specifically, past findings reveal that cognitive empathy in particular is negatively associated with AUD^26^. These observed associations between low empathy and AUD could develop as a result of drinking and then perpetuate the drinking^18^, thereby contributing to the maintenance of AUD and development of interpersonal problems. For example, low cognitive empathy might be explained by the impairment in executive functions caused by alcohol abuse^29^. At the same time, it is also possible that a pre-existing empathy deficit contributes to interpersonal problems and that these increase the risk for AUD. For example, a lack of affective empathy might cause interpersonal distress by an inability to develop and maintain healthy relations, and may therefore lead to drinking alcohol in order to cope^22^.

In line with findings in AUD populations, one meta-analysis that examined non-clinical samples found an association between lower ToM and more alcohol related problems^30^. Additionally, experimental studies in non-AUD individuals have found that drinking alcohol can have acute negative effects on social cognition processes such as facial emotion recognition, empathy and ToM (for a review, see^31^). Consequently, low cognitive empathy (for example, due to a deficit in facial emotion recognition) can contribute to interpersonal distress, and in turn this can contribute to drinking to cope with negative affect associated with the distress^4^.

People have different motives for drinking alcohol. According to Cooper^32^, these drinking motives can be categorized according to their valence (positive, negative) and source (internal, external). This results in four main reasons for drinking: to enhance sociability (external, positive), to enhance positive experiences (internal, positive), to conform to peers (external, negative), and to cope with negative affect (internal, negative). The coping motive is most strongly correlated with heavy drinking and problematic outcomes as shown by cross-sectional studies^33–41^. Moreover, at least one longitudinal study found that drinking to cope predicted alcohol-related problems over time^42^.

Individuals who drink to cope with negative affect might also be prone to drink to cope with others’ pain^43^. Feeling others' pain and processing one’s own pain involves activation of overlapping brain regions^44^. Feeling others’ pain could lead to negative affect in the self that can result in a maladaptive emotional response like drinking to cope with it^45^. Past studies showed a link between negative internal drinking motives (coping with negative affect) and patterns of problematic alcohol use^46^. These problematic patterns of alcohol use may give rise to empathy deficits^19,20^ which, in turn, may lower the threshold for developing interpersonal problems, which again may lower the threshold of alcohol use, etc. In particular, negative drinking motives may contribute to the emergence of alcohol-related issues and a decline in empathy. Individuals who are experiencing interpersonal difficulties may be motivated to drink to overcome these difficulties, which appears to be more closely tied to negative drinking motives (related to experiencing negative affect or a perceived need to conform in social situations) than to positive drinking motives (related to socializing per se or increasing positive affect in social situations). Thus, especially people who drink to cope with negative affect may be at risk of entering this vicious circle and developing interpersonal problems.

### The present study

Previous studies have found that AUD is associated with social cognition deficits, including low empathy^16,17^. The first aim of the present study was to examine whether such an association also exists in a non-clinical sample composed of adults reporting a wide range in alcohol consumption. In agreement with the dimensional perspective on psychopathology, this sample was considered relevant to AUD because it was expected to also include drinkers who would meet the criteria for problematic drinking and therefore be at risk for AUD. As most past studies have been restricted to affective and cognitive empathy, the second aim was to test whether risky alcohol use is also negatively related to behavioral empathy. Thirdly, we examined if the link between risky alcohol use and low empathy is moderated by people’s drinking motives (i.e., if this relationship would be especially pronounced in individuals who drink to cope with negative affect).

The study is novel in that a multimethod approach was used to assess empathy, including both a traditional questionnaire measure for assessing cognitive and affective empathy and a novel measure, involving scenarios of a fictional character experiencing pain and asking participants whether they would help the character, for assessing behavioral empathy. Besides, drinking motives were thus far not examined as a moderator in the alcohol-empathy link.

In light of previous studies^19,21–25^, we hypothesized that: higher alcohol use would be associated with lower levels of both cognitive and affective empathy. Similarly, we expected that higher alcohol use would be associated with lower behavioral empathy. In addition, we hypothesized that the observed associations between alcohol use and empathy would be stronger in people with more negative internal drinking motives in particular (i.e., drinking to cope).

## Methods

### Ethical review

The present study was part of a Double Degree PhD project from Alexandru Ioan Cuza University of Iași, Romania, and the University of Groningen, Netherlands. The study was approved by the relevant Ethics Committees of both universities.

### Participants and procedure

During April and May 2021 we posted advertisements on Facebook that asked for adults located in Romania or the Netherlands to complete an online survey on “Why do people drink alcohol?”. The advertisement included a picture with individuals drinking in a social setting and a picture of someone drinking alone, to prevent self-report bias related to drinking. Additional participants were recruited via the researchers’ personal networks, using a snowballing technique.

Informed consent was provided by 890 out of 1210 individuals who opened the survey link (74%). The survey was completed fully by 520 unselected individuals (42% female). Most participants were 18–25 years old (86%). The preferred language was Romanian for 381 (74%), Dutch for 70 (14%), and English for 63 (12%) individuals.

Individuals who opened the survey link were first asked to indicate their preferred language (Romanian, Dutch, or English) and then continued the survey in that language. After reading a short study description followed by the full study information, participants provided informed consent.

The survey asked for some background information (e.g., gender: male, female, other, prefer not to say; age: < 18, 18–25, 26–35, 36–45, 46–55, 56–65, > 65 years) and included measures of alcohol use, drinking motives, and empathy. Data from an additional measure on corona-related anxiety are not considered in this paper (but see^47^). After responding to all questions, participants were debriefed and had the option to participate in a raffle for a 10 RON/5 euro gift certificate (10% winning rate).

### Measures

To assess hazardous alcohol consumption, we included the *Alcohol Use Disorders Identification Test* [AUDIT^48^]. AUDIT scoring guidelines were respected and an overall total score for every participant was created. The Cronbach coefficient alpha in the present study was 0.85, indicating good internal consistency. Scores ≥ 8 have sufficient sensitivity and specificity to suggest a strong likelihood for risky alcohol use^49^. Accordingly, continuous AUDIT scores (range 0–40) were also recoded into a dichotomous variable to index risky alcohol use.

A simple Quantity—Frequency Measure was used to find out the number of drinks participants consumed on each of the seven weekdays during the last month (US National Institute on Alcohol Abuse and Alcoholism [NIAAA]). Variables subsequently derived included the number of standard drinks consumed per each day of the week (bottles of beer/ glasses of wine/shots of hard liquor), and the frequency of drinking episodes per month.

To assess reasons for drinking alcohol, we administered the *Revised Drinking Motives Questionnaire* [DMQ-R^32^]. Its items load into four factors corresponding with the four drinking motives (drinking to socialize, drinking to cope with negative affect, drinking to enhance positive affect, and drinking to conform to others)*.* Sample items include “You drink because it helps you enjoy a party” (socializing); “You drink to forget your worries” (coping); “You drink to be liked” (conformity), and “You drink because it’s exciting” (enhancement). In the present study, the Cronbach coefficients alpha for these factors ranged between 0.83 and 0.88.

Affective and cognitive empathy were assessed using the *Empathy Quotient* [EQ^12^]. We used the scoring guidelines to create continuous variables for cognitive (the sum score for 11 items) and affective empathy (the sum score of 11 items), resulting in a subscale score ranging between 0 and 22. The subscale social skills was not used. Examples of EQ items are: “I sometimes find it difficult to see things from the ‘other guy’s’ point of view.” (reversely scored, cognitive empathy) and “I am inclined to get nervous when others around me seem to be nervous.” (affective empathy). The Cronbach coefficient alpha of the cognitive empathy subscale was 0.88 and for the affective empathy subscale it was 0.74.

Behavioral empathy was assessed using a novel measure derived from stimuli developed by Bruneau et al.^50^. These stimuli include 4 sets of 12 (written) stories that describe significant emotional or physical pain of a fictional character- subsequently referred to as the high-pain scenarios. Participants were randomized to see one of these four sets and used a 100-point sliding scale to rate their likelihood to help the main character in each scenario (0 = No, I would definitely not help this person, 100 = Yes, I would definitely help this person). There were also 4 sets of 12 control stories that describe comparable scenarios in which there was no (significant) painful outcome—the low-pain scenarios. These were used to measure participants’ general willingness to help others. Findings from Bruneau et al.^50^ indicate that the brain regions associated with the “shared pain network”—including secondary sensory areas, anterior middle cingulate, and bilateral insulae—were more engaged in relation to stories describing high pain scenarios than in relation to stories describing low-pain scenarios. Accordingly, we only considered high pain scenarios a measure of behavioral empathy. Prior to data analysis, we created one mean score for the 12 high-pain scenarios and one for the 12 low-pain scenarios. The mean scores on the sliding scale across the four sets of high pain scenarios were comparable (*F(3,*520*)* = 1.89, *p* = 0.13), as well as for the low pain scenarios (*F(3,*520*)* = 1.93, *p* = 0.12).

### Statistical analyses

SPSS 25 was used to prepare the data for analysis, perform hypothesis testing, and conduct additional analyses. Comparable to aan het Rot et al.^47^, we re-coded gender into 0 = male, 1 = female, and 2 = unknown (the latter category being excluded from data analyses due to the low n), and we re-coded age into 0 = 18–25 years, 1 = 25 + years. Language was considered a covariate because the language subgroups differed on several key variables, see Supplementary Table 1.

To test the first hypothesis regarding the link between risky alcohol use and empathy, we conducted two types of analyses. Firstly, continuous AUDIT scores were entered as a predictor of affective empathy, cognitive empathy, or behavioral empathy in three separate hierarchical linear regressions. Block 1 included the covariates language, binary gender, and binary age category (dummy-coded) and block 2 included the AUDIT scores. Secondly, using analysis of covariance (ANCOVA) and including the same covariates (not dummy coded), dichotomized AUDIT scores were entered as a predictor and the three empathy measures were serially entered as outcomes.

To test the second hypothesis regarding drinking to cope as a moderator in the association between empathy and risky alcohol use AUDIT scores and all four DMQ-R subscales were standardized, and four AUDIT-by-subscale interaction terms were created. In separate hierarchical linear regressions, block 1 included the dummy-coded covariates, block 2 the standardized AUDIT score, block 3 one of the DMQ-R subscales (our primary interest being the coping subscale), block 4 the relevant interaction term. The outcome variable was one of the three empathy measures.

Effect sizes are reported using Cohen’s d values, with d = 0.2 signifying a small effect, d = 0.5 signifying a medium effect, and d = 0.8 signifying a large effect^51^.

### Statement regarding human subjects

All methods were carried out in accordance with relevant guidelines and regulations: The relevant Ethics Committees of University of Groningen and Alexandru Ioan Cuza University of Iași approved the research. Informed consent was obtained from all participants. The study was performed in accordance with the Declaration of Helsinki.

## Results

### Descriptives

The average AUDIT score was 7.8 (*SD* = 0.26), with 39% of participants reporting clinically relevant alcohol problems. Men (*M* = 8.86, *SD* = 6.54) had higher AUDIT scores than women (*M* = 6.39, *SD* = 5.04), *t*(509) = 4.63, *p* < 0.001, *d* = 0.41 (a small to medium effect).

Participants mostly drank to socialize with others (*M* = 13.25, *SD* = 0.22), then to enhance positive affect (*M* = 11.46, *SD* = 0.21), then to cope with negative affect (*M* = 9.27, *SD* = 0.20), and then to conform to others (*M* = 6.67, *SD* = 0.12). The average score on affective empathy was 10.53 (*SD* = 0.17) and for cognitive empathy it was 12.03 (*SD* = 0.19).

Participants were generally more likely to help others in the high-pain scenarios (thought to indicate behavioral empathy) than in the low-pain scenarios (thought to indicate general helping behavior), *t*(519) = 33.12, *p* < 0.001, *d* = 2.90 (a very large effect). The average score on behavioral empathy was *M* = 78.47 (*SD* = 15.79) and the average score on general helping behavior was *M* = 42.58 (*SD* = 21.17). The correlation between behavioral empathy and general helping was positive but weak (*r* = 0.130, *p* = 0.003). In support of the validity of our novel measure, behavioral empathy correlated moderately positively with both EQ subscales (cognitive *r* = 0.26, *p* < 0.0001, and affective *r* = 0.40, *p* < 0.0001), while the correlation between general helping and the cognitive empathy subscale was only weakly positive (*r* = 0.13, *p* = 0.001) and the correlation between general helping and the affective empathy subscale was not significant (*r* = 0.07, *p* = 0.12).

### Preliminary analyses

Continuous AUDIT scores were positively associated with all four drinking motives (all *p’s* < 0.001; see Supplementary Table 2a for details). Similarly, participants with risky drinking (based on dichotomized AUDIT scores) were more likely to endorse all four drinking motives than participants with low drinking levels (see Supplementary Table 3a). The largest effect sizes were found for drinking to cope.

Affective empathy was not significantly associated with any drinking motive (all *p’s* > 0.16, see Supplementary Table 4a). Cognitive empathy was positively associated with drinking to enhance, *p* < 0.05, but was not significantly associated with socializing, coping, or conformity, (*p’s* > 0.24). Behavioral empathy was negatively associated with drinking to cope (*p* = 0.02) and drinking to conform (*p* < 0.001) and not significantly associated with socializing (*p* = 0.55) or enhancing (*p* = 0.30). General helping behavior was not significantly associated with any drinking motive (all *p’s* > 0.10, see Supplementary Table 4a). Although these regression results point towards a relation between cognitive empathy and positive drinking motives vs. between behavioral empathy and negative drinking motives, a similar differential pattern was not found in the ANCOVA results (see Supplementary Table 3a).

### Hypothesis 1: Alcohol use and empathy

Continuous AUDIT scores did not significantly predict affective empathy (*p* = 0.15, *d* = 0.12), cognitive empathy (*p* = 0.53, *d* = 0.05), or general helping behavior (*p* = 0.42, *d* = 0.06) but did negatively predict behavioral empathy, p < 0.001, with a small to medium effect size, *d* = 0.30 (see Table 1 for detailed statistics). When correcting for multiple testing and adjusting the alpha to 0.05/4 = 0.0125, the effect for BE remained significant. In comparison, dichotomized AUDIT scores did not significantly relate to any of the four outcome variables (See Supplementary Table 3a).

**Table 1:** AUDIT by empathy measures.

Outcomes	Predictors	∆F	∆R^2^	b	t
Affective empathy	Block 1	33.22***	0.16		
	Gender			0.36	8.75***
	Age			− 0.004	− 1.05
	Romanian			− 0.12	− 2.82**
	Block 2	2.03	0.003		
	Gender			0.35	8.28***
	Age			− 0.04	− 0.95
	Romanian			− 0.12	− 2.91**
	AUDIT Score			− 0.06	− 1.42
Cognitive empathy	Block 1	6.67***	0.04		
	Gender			0.18	4.03***
	Age			− 0.06	− 1.42
	Romanian			0.02	0.59
	Block 2	0.37	0.001		
	Gender			0.17	3.82***
	Age			− 0.06	− 1.38
	Romanian			0.02	0.55
	AUDIT score			− 0.02	− 0.61
Behavioral empathy	Block 1	12.27***	0.06		
	Gender			0.23	5.39***
	Age			− 0.05	− 1.25
	Romanian			− 0.06	− 1.31
	Block 2	12.14***	0.02		
	Gender			0.20	4.62***
	Age			− 0.04	− 1.02
	Romanian			− 0.07	− 1.54
	AUDIT score			− 0.15	− 3.48***
General willingness to help	Block 1	10.27***	0.05		
	Gender			0.03	0.69
	Age			0.11	2.42
	Romanian			0.18	4.02***
	Block 2	0.63	0.001		
	Gender			0.02	0.51
	Age			0.10	2.36
	Romanian			0.18	3.96***
	AUDIT scores			− 0.03	− 0.79

*p < 0.05, **p < 0.01, ***p < 0.001. N = 520. Romanian was coded with 3 as the third option from the Preferred language variable.

### Hypothesis 2: Drinking to cope as a moderator of the links between drinking and empathy

As continuous AUDIT scores were negatively associated with behavioral empathy alone (see results for Hypothesis 1) we conducted moderator analyses for this outcome variable only.

**Figure 1:**
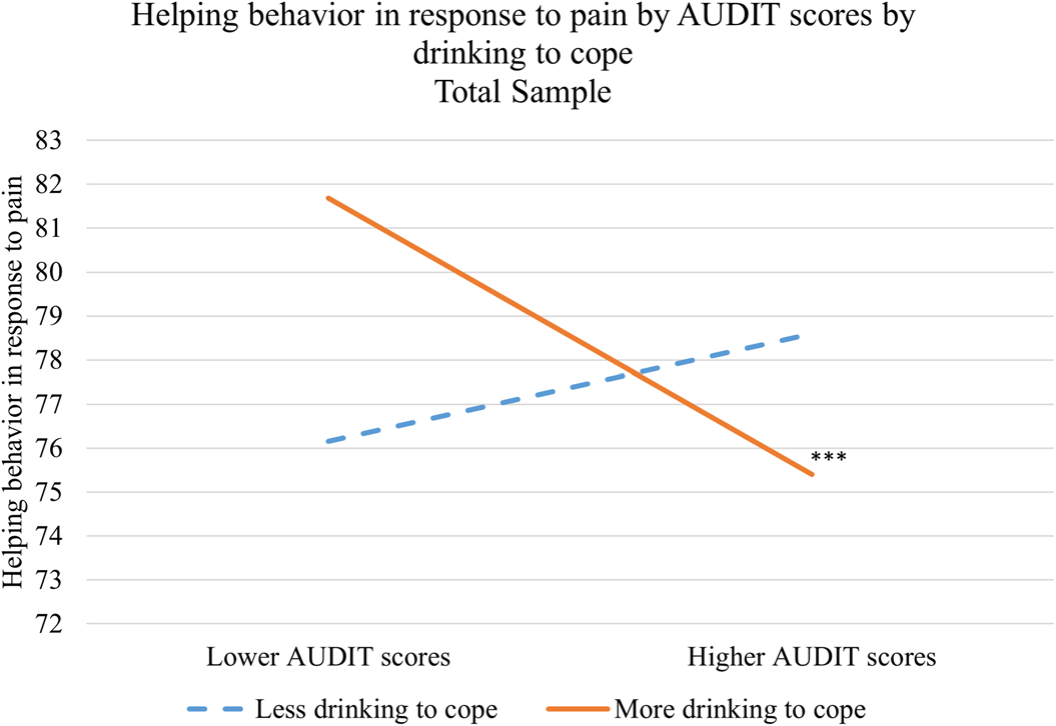
Drinking to cope as moderator between drinking and behavioral empathy association. ***p < 0.001, representing the negative association found among individuals less likely to drink to cope (b =  − 1.66).

The drinking to cope by AUDIT interaction was significant, *b* = − 0.19, *t*(504) = − 3.55, *p* < 0.001, *d* = 0.31. Post-hoc tests revealed that participants who were more likely to drink to cope with negative affect showed a significant negative association between AUDIT scores and behavioral empathy, *b* = − 2.18, *p* < 0.001 whereas participants who were less likely to drink to cope did not, *b* = 0.59, *p* = 0.48, see Fig. 1.

The negative association between continuous AUDIT scores and behavioral empathy was also moderated by drinking to conform, as indicated by a significant interaction term, *b* = − 0.13, *t*(504) = − 2.82, *p* < 0.01, *d* = 0.25. Participants who were more likely to drink to conform showed a significant negative association, *b* = − 1.66, *p* < 0.05, whereas participants who were less likely to drink to conform did not, *b* = − 0.25, *p* = 0.73.

To examine if the moderating effect of drinking to cope would be independent of drinking to conform, we repeated the first regression analysis with drinking to conform added as a control variable. The drinking to cope by AUDIT interaction remained significant *b* = − 0.19, *t*(503) = − 3.53, *p* < 0.001, *d* = 0.31. We also repeated the second regression, adding drinking to cope as a control variable; the drinking to conform by AUDIT interaction term remained significant *b* = − 0.13, *t*(503) = − 2.80, *p* < 0.05, *d* = 0.25.

### Sensitivity analyses in the Romanian subsample

In the Romanian subgroup 36% of participants scored above the clinical cut-off of the AUDIT, which is comparable to the total sample. Outcomes of hypothesis testing are also comparable, except (a) continuous AUDIT scores also predicted lower affective empathy, *p* < 0.01, see Table 2, and (b) additional significant ANCOVA results indicated that Romanian risky drinkers scored lower on affective empathy, *p* < 0.01, and on general helping behavior, *p* < 0.05, than normal drinkers, see Supplementary Table 3b.

**Table 2:** AUDIT by empathy measures in the Romanian subsample.

Outcomes	Predictors	∆F	∆R^2^	b	t
Affective empathy	Block 1	32.56***	0.14		
	Gender			0.37	7.69***
	Age			− 0.07	− 1.61
	Block 2	7.52	0.1		
	Gender			0.35	7.17***
	Age			0.07	1.35
	AUDIT Score			− 0.13	− 2.74**
Cognitive empathy	Block 1	12.40***	0.06		
	Gender			0.21	4.23***
	Age			− 0.10	− 2.15
	Block 2	1.67	0.004		
	Gender			0.20	3.95***
	Age			− 0.10	− 2.02
	AUDIT score			− 0.06	− 1.29
Behavioral empathy	Block 1	11.90***	0.50		
	Gender			0.22	4.38***
	Age			− 0.08	− 1.67
	Block 2	17.74***	0.40		
	Gender			0.18	3.68***
	Age			− 0.06	− 1.30
	AUDIT score			− 0.21	− 4.21***
General willingness to help	Block 1	3.70	0.02		
	Gender			0.06	1.24
	Age			0.11	0.28
	Block 2	1.23	0.003		
	Gender			0.05	1.03
	Age			0.11	2.16
	AUDIT scores			− 0.06	− 1.11

*p < 0.05, **p < 0.01, ***p < 0.001. N = 387.

**Figure 2:**
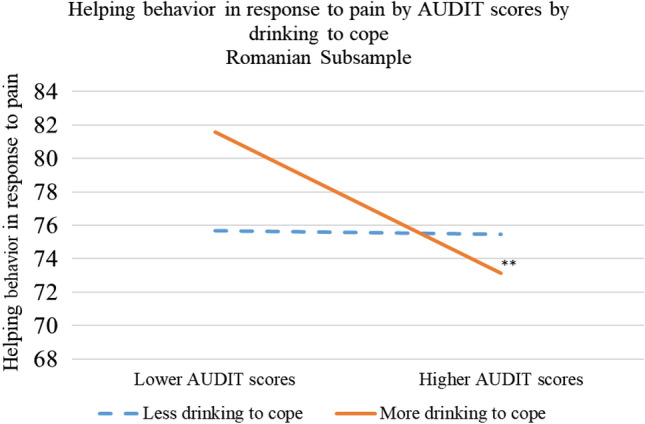
Drinking to cope as moderator between drinking and behavioral empathy association in the Romanian subsample. *p < 0.05, **p < 0.01, ***p < 0.001.

Most importantly, for hypothesis 2, we again found that the negative association between AUDIT and behavioral empathy was stronger for participants who were more likely to drink to cope with negative affect, *b* = − 0.18, *t*(379) = − 2.72, *p* < 0.05, see Fig. 2.

## Discussion

The present study examined the relations between (problematic) alcohol use and affective, cognitive, and behavioral empathy in a non-clinical sample. The moderating role of drinking motives was also examined.

The first finding was that drinking more was primarily associated with less behavioral empathy, and not with lower affective or cognitive empathy. This outcome is consistent with correlational studies on long term drinking, that found complementary lower trait cognitive empathy in people with AUD^26^, lower ToM ability and lowered performance on facial emotion recognition tasks^16^. These outcomes support the idea that long term problematic drinking is associated with impairments in trait cognitive empathy, and related problematic outcomes^30^.

According to the You and Chou^52^ model regarding neurocognitive pathways of affective, cognitive and behavioral empathy, behavioral empathy is the result of both cognitive and affective empathy but on different routes. Precisely, the affective empathy component manifests on a fast route were sensory information is rapidly transmitted to the amygdala from the thalamus; on the other side the cognitive component manifests on a slow route were higher level consciousness processing takes place from the visual pathway to the retinal neurons and visual cortex^52^. Both routes concretize into prosocial behavior, specifically, helping that is seemingly lower in people that drink more. Thereby the resulting lower behavioral empathy might be associated with more drinking early on, suggesting that cognitive and affective pathways might become altered by alcohol in time^31^. Past studies have found both affective^22^ and cognitive empathy to be low in people with AUD^26^. Besides, one previous experimental study^53^ found an acute alcohol effect on the neural correlates responsible for behavioral empathy (empathy for pain). On the one hand, acute alcohol effects of alcohol on behavioral empathy may also be associated with interpersonal problems that maintain the drinking. On the other hand, a lack of affective empathy might cause interpersonal distress, and may therefore lead to drinking alcohol in order to cope^22^. Drinking more may be associated with other interpersonal problems specifically caused by affected behavioral empathy. These are two possible mechanisms that might explain the development of AUD, namely interpersonal problems that people with AUD have might be explained by deficits in empathy.

At the same time, it appears that behavioral empathy may be reduced even at lower drinking levels. As cognitive empathy is thought to contribute to behavioral empathy, rather than the other way around^54^, this was a surprising result. However, our novel behavioral empathy measure may be more sensitive in picking up empathy deficits than the EQ, thanks to its use of vignettes about how people might behave in a given situation (state empathy) rather than asking for more generic/habitual behaviors (trait empathy) as is done in the EQ. Indeed, experimental studies have shown that even acute alcohol consumption can (but does not always) impair empathy and related aspects of social cognition as measured using computer tasks and exposure to emotional or painful images^55^.

### Drinking to cope with negative affect elicited by others?

People who were more likely to drink to cope with negative affect had a stronger association between drinking and low behavioral empathy. In line with this, drinking to cope with one’s depressive feelings has previously been linked to low self-compassion^56^ and low self-compassion to low empathy^57^. Low self compassion may therefore also hamper the inclination of helping others. Thus, drinking to cope with one’s own negative affect might be understood as an attempt to help oneself which in turn might be connected to a reduced capacity to help others.

The present results are particularly relevant in light of the sample characteristics: young adult drinkers who report problematic alcohol use in a percentage of 39%, were included in the study after they have responded to social media advertisements. As problematic alcohol drinking increases the risk of developing AUD the results might have clinical relevance. Namely, people who drink now to cope with negative affect elicited by others' pain, have a negative impacted helping behavior, consequently the drinking might become problematic, thus developing into AUD later in life^58^. Further prolonged alcohol use might diminish cognitive empathy^53^ thereby promoting the development of a vicious cycle: consuming alcohol to cope with negative affect contributes to interpersonal problems (low behavioral empathy) which in turn may lower the threshold for engaging in (risky) drinking behavior^4^.

Cultural factors might have influenced the observed association between alcohol use and empathy. At a cultural level people living in Romania exhibit a relatively high propensity for avoiding uncertainty compared to people living in a western European culture^59^. Relatedly, previous research found support for the idea that Romanians may respond slightly differently to others’ negative emotional expressions than the Dutch^60^. People in Romania may thus possess a diminished ability to manage unpleasant situations^59^, leading them to be more likely to show diminished empathy after drinking alcohol.

### Limitations and strengths

The cross-sectional nature of the study has limitations. Specifically, it precludes the drawing of conclusions about the direction of the observed relations between the studied variables. To examine whether alcohol use may contribute to reductions in (behavioral) empathy over time, longitudinal studies are more suitable. Additionally, experimental studies may examine acute effects of drinking on (behavioral) empathy^55^. Future research should also differentiate when measuring trait or state empathy and include measures that reflect both components.

While previous studies have found an association between AUD and cognitive and affective empathy, in our sample we did not find such associations. While these past studies have also used the EQ to assess affective and cognitive empathy, they used clinical interviews to establish AUD and not the AUDIT. The null findings in the present study regarding cognitive and affective empathy and alcohol associations might be due to the method used to assess problematic alcohol use^61^. Cultural factors might have influenced the generalizability of the results of this study to some degree, and further research is needed in different samples (e.g., Western European, American) to arrive at a more firm conclusion.

The main strong point of the present study is the use of different measures to assess different forms of empathy (cognitive, affective, and behavioral). We only found an association between alcohol use and behavioral empathy (and not between alcohol use and cognitive or affective empathy). This might be explained by the use of different measures, namely the EQ versus a novel empathy measure asking participants about helping others in painful situations. The EQ asks broad questions about how a person might be in general, while our BE measure asks individuals to imagine themselves in specific situations and then asks them about their behavior in these specific situations. Another strength is the use of standardized measures such as the AUDIT and the DMQ-R to assess alcohol use and drinking motives.

To conclude, cross-sectionally alcohol use was negatively associated with behavioral empathy, particularly among participants who were more likely to drink to cope with negative affect. This points towards the idea that not only one’s own negative affect matters, but also that of others. If these results were confirmed in longitudinal studies, this could help the development of new AUD prevention strategies.

### Supplementary Information

Supplementary Information.

## Data Availability

The datasets generated and analyzed during the current study will be available by the time of the publication of the paper in the DataverseNL repository, https://doi.org/10.34894/KK33YT.
