# Supplementary material for: Associations between alcohol consumption and empathy in a non-clinical sample: drinking motives as a moderator
Source: Sci Rep. 2024 May 14;14:10993. doi: 10.1038/s41598-024-59233-x (PMC11094050; doi:10.1038/s41598-024-59233-x)
Supplement: Supplementary file 1 — Supplementary Information. [file 41598_2024_59233_MOESM1_ESM.docx]

**Associations between alcohol consumption and empathy in a non-clinical sample:**

**Drinking motives as a moderator**

Tables and figures

Isabelle C. Baltariu, MSc^1,2^*, Violeta Enea, PhD^2^, Peter J. de Jong, PhD^1^, Marije aan het Rot, PhD^1^

*^1^Department of Psychology, University of Groningen, The Netherlands; ^2^Department of Psychology, Alexandru Ioan Cuza University of Iași, Romania*

S1. Supplementary Table 1. Descriptive data

|  | | **Romanian N=387** | **Dutch N=70** | **English N=63** | **Subgroup Differences** |
| --- | --- | --- | --- | --- | --- |
| **Gender** | **Female %** | 37 | 43 | 75 | X^2^(2) = 31.09*** |
|  | **Male %** | 63 | 57 | 25 |  |
| **Age** | **18-25 years %** | 65 | 79 | 54 | X^2^(2) = 50.78*** |
|  | **25+ years %** | 35 | 21 | 46 |  |
| **AUDIT** | **Total score, M(SD)** | 7.63(6.16) | 8.35(5.51) | 8.19(5.94) | F(2,520)=0.57 |
|  | **Clinical score %** | 36 | 47 | 46 |  |
| **EQ** | **Affective, M(SD)** | 10.09(3.79)^a^ | 11.45(3.63)^b^ | 12.15(4.69)^c^ | F(2,520)=9.86***^ab, ac,^ |
|  | **Cognitive, M(SD)** | 12.06(4.44) | 12.21(3.85) | 12.15(4.69) | F(2,520)=0.27 |
| **Scenarios** | **Low pain, M(SD)** | 45.29(21.2)^a^ | 32.66(17.13)^b^ | 36.93(20.94)^c^ | F(2,520)=13.74***^ab, ac^ |
|  | **High pain, M(SD)** | 77.69(16.86) | 79.14(11.70) | 82.53(12.05) | F(2,520)=2.62 |
| **DMQR** | **Socializing, M(SD)** | 13.29(5.12) | 12.27(4.66) | 14.14(5.17) | F(1,520)=2.29 |
|  | **Enhancing, M(SD)** | 11.17(5.12)^a^ | 11.42(4.17)^b^ | 13.33(4.67)^c^ | F(1,520)=5.15**^ac^ |
|  | **Coping, M(SD)** | 9.47(4.95)^a^ | 7.55(2.93)^b^ | 9.95(4.33)^c^ | F(1,520)=5.78**^cb,ba^ |
|  | **Conformity, M(SD)** | 6.76(3.14) | 5.92(1.6) | 6.95(2.66) | F(520)=2.75 |

Note. AUDIT=Alcohol Use Disorder Identification Test, EQ= The Empathy Quotient Questionnaire, DMQ-R=Revised Drinking Motives Questionnaire, M=mean, SD=standard deviation. *p<0.05 **p<0.01 ***p<0.001. ^a,b,c^the results for the Omnibus test regarding subgroup differences

S2. Supplementary Table 2a. AUDIT by drinking motives.

| **Outcomes** | **Predictors** | **∆F** | **∆R^2^** | **b** | **t** |
| --- | --- | --- | --- | --- | --- |
| **DMQR Socializing** | **Block 1** | 7.53*** | .04 |  |  |
|  | **Gender** |  |  | -.05 | -1.20 |
|  | **Age** |  |  | -.21 | -4.70*** |
|  | **Romanian** |  |  | -.06 | -1.03 |
|  | **Block 2** | 108.08*** | .16 |  |  |
|  | **Gender** |  |  | .03 | 0.82 |
|  | **Age** |  |  | -.24 | -5.86*** |
|  | **Romanian** |  |  | -.03 | -0.78 |
|  | **AUDIT Score** |  |  | .42 | 10.39*** |
| **DMQR Coping** | **Block 1** | 2.08 | .01 |  |  |
|  | **Gender** |  |  | .07 | 1.54 |
|  | **Age** |  |  | .05 | 1.06 |
|  | **Romanian** |  |  | .10 | 2.21* |
|  | **Block 2** | 298.22*** | .36 |  |  |
|  | **Gender** |  |  | .19 | 5.44*** |
|  | **Age** |  |  | .00 | 0.16 |
|  | **Romanian** |  |  | .14 | 3.85*** |
|  | **AUDIT score** |  |  | .62 | 17.26*** |
| **DMQR Enhancing** | **Block 1** | 4.46* | .02 |  |  |
|  | **Gender** |  |  | -.02 | -0.58 |
|  | **Age** |  |  | -.13 | -2.96* |
|  | **Romanian** |  |  | -.13 | -2.94* |
|  | **Block 2** | 216.68*** | .29 |  |  |
|  | **Gender** |  |  | .9 | 2.33 |
|  | **Age** |  |  | -.17 | -4.52* |
|  | **Romanian** |  |  | -.1 | -2.59* |
|  | **AUDIT score** |  |  | .55 | 14.72*** |
| **DMQR Conformity** | **Block 1** | 0.83 | .005 |  |  |
|  | **Gender** |  |  | -.003 | -0.07 |
|  | **Age** |  |  | -.05 | -1.11 |
|  | **Romanian** |  |  | .03 | 0.73 |
|  | **Block 2** | 63.16*** | .11 |  |  |
|  | **Gender** |  |  | .06 | 1.55 |
|  | **Age** |  |  | -.07 | -1.71 |
|  | **Romanian** |  |  | .05 | 1.26 |
|  | **AUDIT scores** |  |  | 0.34 | 7.94*** |

Note. *p<0.05 **p<0.01 ***p<0.001. N=520.S3. Supplementary Table 2b. AUDIT by drinking motives in the Romanian subsample.

| **Outcomes** | **Predictors** | **∆F** | **∆R^2^** | **b** | **t** |
| --- | --- | --- | --- | --- | --- |
| **DMQR Socializing** | **Block 1** | 6.76*** | .03 |  |  |
|  | **Gender** |  |  | -.7 | -1.55 |
|  | **Age** |  |  | -.17 | -3.48*** |
|  | **Block 2** | 81.72*** | .17 |  |  |
|  | **Gender** |  |  | -.006 | -0.12 |
|  | **Age** |  |  | -.21 | -4.64*** |
|  | **AUDIT Score** |  |  | .42 | 9.04*** |
| **DMQR Coping** | **Block 1** | 2.84* | .01 |  |  |
|  | **Gender** |  |  | .08 | 1.64 |
|  | **Age** |  |  | .09 | 1.89 |
|  | **Block 2** | 244.86*** | .38 |  |  |
|  | **Gender** |  |  | .19 | 4.78*** |
|  | **Age** |  |  | .04 | 1 |
|  | **AUDIT score** |  |  | .63 | 15.64*** |
| **DMQR Enhancing** | **Block 1** | 1.3 | .007 |  |  |
|  | **Gender** |  |  | -.04 | -0.87 |
|  | **Age** |  |  | -.07 | -1.44 |
|  | **Block 2** | 154.12*** | .28 |  |  |
|  | **Gender** |  |  | .05 | 1.12 |
|  | **Age** |  |  | -.12 | -2.83 |
|  | **AUDIT score** |  |  | .54 | 12.41*** |
| **DMQR Conformity** | **Block 1** | 0.08 | 0 |  |  |
|  | **Gender** |  |  | -.004 | -0.06 |
|  | **Age** |  |  | -.02 | -0.39 |
|  | **Block 2** | 60.23*** | .13 |  |  |
|  | **Gender** |  |  | .06 | 1.26 |
|  | **Age** |  |  | -.05 | -1.13 |
|  | **AUDIT scores** |  |  | .37 | 7.76*** |

Note. *p<0.05 **p<0.01 ***p<0.001. N=387.

S4. Supplementary Table 3a. The association between harmful alcohol use and empathy, and between harmful alcohol use and drinking motives.

|  | | **Low AUDIT**  **(N=318)** | **High AUDIT**  **(N=202)** |  |  |
| --- | --- | --- | --- | --- | --- |
| **EQ** | **Affective empathy** | 11.37 (0.26) | 10.88 (0.29) | F(1,510)=2.15 | d=0.12 |
|  | **Cognitive empathy** | 12.06(0.32) | 11.82(0.36) | F(1,510)=0.34 | d=0.05 |
| **Scenarios** | **Behavioral empathy** | 80.56(1.12) | 78.95(1.23) | F(1,510)=1.29 | d=0.10 |
|  | **General helping behavior** | 40.21(1.49) | 36.80(1.64) | F(1,510)=3.23 | d=0.15 |
| **DMQR** | **Socializing** | 11.55(0.33) | 15.74(0.36) | F(1,510)=97.89*** | d=0.85 |
|  | **Coping** | 6.82(0.29) | 11.70(0.32) | F(1,510)=169.41*** | d=1.15 |
|  | **Enhancing** | 9.95(0.32) | 14.79(0.35) | F(1,510)=143.19*** | d=1.04 |
|  | **Conforming** | 5.86(0.20) | 7.50(0.22) | F(1,510)=38.75*** | d=0.54 |

Note. Scores are estimated marginal means (SE) included into ANCOVA analysis including language, gender and age as covariates. AUDIT=Alcohol Use Disorder Identification Test, Low AUDIT scores were >8, High AUDIT scores were <8. EQ= The Empathy Quotient Questionnaire, DMQ-R=Revised Drinking Motives Questionnaire, SE=standard error. *p<0.05 **p<0.01 ***p<0.001. N=520

S5. Supplementary Table 3b. The association between harmful alcohol use and empathy, and between harmful alcohol use and drinking motives in the Romanian subsample.

|  | | **Low AUDIT**  **(N=242)** | **High AUDIT**  **(N=138)** |  |  |
| --- | --- | --- | --- | --- | --- |
| **EQ** | **Affective empathy** | 10.71(0.23) | 9.75(0.31) | F(1,379)=6.44** | d=0.26 |
|  | **Cognitive empathy** | 12.31(0.29) | 11.82(0.39) | F(1,379)=0.29 | d=0.05 |
| **Scenarios** | **Behavioral empathy** | 79.28(1.10) | 76.18(1.47) | F(1,379)=3.07 | d=0.18 |
|  | **General helping behavior** | 46.34(1.40) | 41.53(1.88) | F(1,379)=4.58* | d=0.21 |
| **DMQR** | **Socializing** | 10.49(0.41) | 14.97(0.48) | F(1,379)=81.23*** | d=0.92 |
|  | **Coping** | 8.13(0.37) | 13.58(0.44) | F(1,379)=142.72*** | d=1.22 |
|  | **Enhancing** | 8.76(0.4) | 14.05(0.47) | F(1,379)=118.73*** | d=1.11 |
|  | **Conforming** | 5.93(0.27) | 7.97(0.31) | F(1,379)=39.32*** | d=0.64 |

Note. Scores are estimated marginal means (SE) included into ANCOVA analysis including language, gender and age as covariates. AUDIT=Alcohol Use Disorder Identification Test, Low AUDIT scores were >8, High AUDIT scores were <8. EQ= The Empathy Quotient Questionnaire, DMQ-R=Revised Drinking Motives Questionnaire, SE=standard error. *p<0.05 **p<0.01 ***p<0.001. N=387

S6. Supplementary Table 4a. Empathy by drinking motives.

| **Outcomes** | **Predictors** | **∆F** | **∆R^2^** | **b** | **t** |
| --- | --- | --- | --- | --- | --- |
| **DMQR Socializing** | **Block 1** | 7.53** | .04 |  |  |
|  | **Gender** |  |  | -.05 | -1.20 |
|  | **Age** |  |  | -.21 | -4.70*** |
|  | **Romanian** |  |  | -.06 | -1.30 |
|  | **Block 2** | 1.02 | .002 |  |  |
|  | **Gender** |  |  | -.07 | -1.49 |
|  | **Age** |  |  | -.21 | -4.64*** |
|  | **Romanian** |  |  | -.05 | -1.16 |
|  | **EQ Affective** |  |  | .04 | 1.01 |
| **DMQR Coping** | **Block 1** | 2.08 | .01 |  |  |
|  | **Gender** |  |  | .07 | 1.54 |
|  | **Age** |  |  | .05 | 1.06 |
|  | **Romanian** |  |  | .10 | 2.21* |
|  | **Block 2** | 1.39 | .003 |  |  |
|  | **Gender** |  |  | .09 | 1.86 |
|  | **Age** |  |  | .04 | 1.01 |
|  | **Romanian** |  |  | .09 | 2.05* |
|  | **EQ Affective** |  |  | -.05 | -1.17 |
| **DMQR Enhancing** | **Block 1** | 4.46* | .02 |  |  |
|  | **Gender** |  |  | -.02 | -0.58 |
|  | **Age** |  |  | -.13 | -2.96* |
|  | **Romanian** |  |  | -.13 | -2.94* |
|  | **Block 2** | 0.18 | .000 |  |  |
|  | **Gender** |  |  | -.03 | -0.69 |
|  | **Age** |  |  | -.13 | -2.93* |
|  | **Romanian** |  |  | -.13 | -2.86* |
|  | **EQ Affective** |  |  | .02 | 0.42 |
| **DMQR Conforming** | **Block 1** | 0.83 | .005 |  |  |
|  | **Gender** |  |  | -.003 | -0.07 |
|  | **Age** |  |  | -.05 | -1.11 |
|  | **Romanian** |  |  | .03 | 0.73 |
|  | **Block 2** | 2.08 | .004 |  |  |
|  | **Gender** |  |  | -.02 | -0.45 |
|  | **Age** |  |  | -.05 | -1.17 |
|  | **Romanian** |  |  | .02 | 0.55 |
|  | **EQ affective** |  |  | -.07 | -1.44 |
| **DMQR Socializing** | **Block 1** | 7.53** | .04 |  |  |
|  | **Gender** |  |  | -.05 | -1.20 |
|  | **Age** |  |  | -.21 | -4.70*** |
|  | **Romanian** |  |  | -.06 | -1.30 |
|  | **Block 2** | 1.11 | .002 |  |  |
|  | **Gender** |  |  | -.06 | -1.37 |
|  | **Age** |  |  | -.21 | -4.62*** |
|  | **Romanian** |  |  | -.06 | -1.32 |
|  | **EQ Cognitive** |  |  | .04 | 1.05 |
| **DMQR Coping** | **Block 1** | 2.08 | .01 |  |  |
|  | **Gender** |  |  | .07 | 1.54 |
|  | **Age** |  |  | .05 | 1.06 |
|  | **Romanian** |  |  | .10 | 2.21* |
|  | **Block 2** | 0.25 | .001 |  |  |
|  | **Gender** |  |  | .07 | 1.60 |
|  | **Age** |  |  | .04 | 1.03 |
|  | **Romanian** |  |  | .10 | 2.22* |
|  | **EQ Cognitive** |  |  | -.02 | 0.50 |
| **DMQR Enhancing** | **Block 1** | 4.46* | .02 |  |  |
|  | **Gender** |  |  | -.02 | -0.58 |
|  | **Age** |  |  | -.13 | -2.96* |
|  | **Romanian** |  |  | -.13 | -2.94* |
|  | **Block 2** | 3.38* | .006 |  |  |
|  | **Gender** |  |  | -.04 | -0.89 |
|  | **Age** |  |  | -.13 | -2.84* |
|  | **Romanian** |  |  | -.14 | -2.99* |
|  | **EQ Cognitive** |  |  | .08 | 1.83* |
| **DMQR Conforming** | **Block 1** | 0.83 | .005 |  |  |
|  | **Gender** |  |  | -.003 | -0.07 |
|  | **Age** |  |  | -.05 | -1.11 |
|  | **Romanian** |  |  | .03 | 0.73 |
|  | **Block 2** | 0.40 | .001 |  |  |
|  | **Gender** |  |  | -.002 | -0.03 |
|  | **Age** |  |  | -.05 | -1.14 |
|  | **Romanian** |  |  | .03 | 1.75 |
|  | **EQ Cognitive** |  |  | -.02 | -0.63 |
| **DMQR Socializing** | **Block 1** | 7.53** | .04 |  |  |
|  | **Gender** |  |  | -.05 | -1.20 |
|  | **Age** |  |  | -.21 | -4.70*** |
|  | **Romanian** |  |  | -.06 | -1.30 |
|  | **Block 2** | 0.22 | .000 |  |  |
|  | **Gender** |  |  | -.05 | -1.28 |
|  | **Age** |  |  | -.21 | -4.66*** |
|  | **Romanian** |  |  | -.05 | -1.26 |
|  | **High pain scenarios** |  |  | .02 | 0.47 |
| **DMQR Coping** | **Block 1** | 2.08 | .01 |  |  |
|  | **Gender** |  |  | .07 | 1.54 |
|  | **Age** |  |  | .05 | 1.06 |
|  | **Romanian** |  |  | .10 | 2.21* |
|  | **Block 2** | 4.57* | .009 |  |  |
|  | **Gender** |  |  | .09 | 2.00* |
|  | **Age** |  |  | .04 | 0.95 |
|  | **Romanian** |  |  | .09 | 2.09* |
|  | **High pain scenarios** |  |  | -.09 | -2.13* |
| **DMQR Enhancing** | **Block 1** | 4.46* | .02 |  |  |
|  | **Gender** |  |  | -.02 | -0.58 |
|  | **Age** |  |  | -.13 | -2.96* |
|  | **Romanian** |  |  | -.13 | -2.94* |
|  | **Block 2** | 1.22 | .002 |  |  |
|  | **Gender** |  |  | -.01 | -0.30 |
|  | **Age** |  |  | -.13 | -3.01* |
|  | **Romanian** |  |  | -.14 | -3.00* |
|  | **High pain scenarios** |  |  | -.05 | -1.10 |
| **DMQR Conformity** | **Block 1** | 0.83 | .005 |  |  |
|  | **Gender** |  |  | -.003 | -0.07 |
|  | **Age** |  |  | -.05 | -1.11 |
|  | **Romanian** |  |  | .03 | 0.73 |
|  | **Block 2** | 6.62** | .01 |  |  |
|  | **Gender** |  |  | .02 | 0.53 |
|  | **Age** |  |  | -.05 | -1.25 |
|  | **Romanian** |  |  | .02 | 0.59 |
|  | **High pain scenarios** |  |  | -.11 | -2.57** |
| **DMQR Socializing** | **Block 1** | 7.53** | .04 |  |  |
|  | **Gender** |  |  | -.05 | -1.20 |
|  | **Age** |  |  | -.21 | -4.70*** |
|  | **Romanian** |  |  | -.06 | -1.30 |
|  | **Block 2** | 4.83 | .009 |  |  |
|  | **Gender** |  |  | -.05 | -1.14 |
|  | **Age** |  |  | -.22 | -4.92*** |
|  | **Romanian** |  |  | .04 | 0.89 |
|  | **Low pain scenarios** |  |  | -.09 | -2.19 |
| **DMQR Coping** | **Block 1** | 2.08 | .01 |  |  |
|  | **Gender** |  |  | .07 | 1.54 |
|  | **Age** |  |  | .05 | 1.07 |
|  | **Romanian** |  |  | .10 | 2.22* |
|  | **Block 2** | 1.07 | .002 |  |  |
|  | **Gender** |  |  | .07 | 1.57 |
|  | **Age** |  |  | .04 | 0.95 |
|  | **Romanian** |  |  | .11 | 2.36* |
|  | **Low pain scenarios** |  |  | -.05 | -1.03 |
| **DMQR Enhancing** | **Block 1** | 4.46* | .02 |  |  |
|  | **Gender** |  |  | -.02 | -0.58 |
|  | **Age** |  |  | -.13 | -2.96* |
|  | **Romanian** |  |  | -.13 | -2.94* |
|  | **Block 2** | 2.27 | .004 |  |  |
|  | **Gender** |  |  | -.02 | -0.53 |
|  | **Age** |  |  | -.14 | -3.10* |
|  | **Romanian** |  |  | -.12 | -2.63* |
|  | **Low pain scenarios** |  |  | -.06 | -1.50 |
| **DMQR Conformity** | **Block 1** | 0.83 | .005 |  |  |
|  | **Gender** |  |  | -.003 | -0.07 |
|  | **Age** |  |  | -.05 | -1.11 |
|  | **Romanian** |  |  | .03 | 0.73 |
|  | **Block 2** | 0.27 | .001 |  |  |
|  | **Gender** |  |  | -.004 | -0.09 |
|  | **Age** |  |  | -.04 | -1.04 |
|  | **Romanian** |  |  | .03 | 0.63 |
|  | **Low pain scenarios** |  |  | .02 | 0.52 |

Note. *p<0.05 **p<0.01 ***p<0.001. N=520.

S7. Supplementary Table 4b. Empathy by drinking motives in the Romanian subsample.

| **Outcomes** | **Predictors** | **∆F** | **∆R^2^** | **b** | **t** |
| --- | --- | --- | --- | --- | --- |
| **DMQR Socializing** | **Block 1** | 6.76*** | .03 |  |  |
|  | **Gender** |  |  | -.7 | -1.55 |
|  | **Age** |  |  | -.17 | -3.48*** |
|  | **Block 2** | 0 | .17 |  |  |
|  | **Gender** |  |  | -.07 | -1.27 |
|  | **Age** |  |  | -.17 | -3.48*** |
|  | **EQ Affective** |  |  | -.02 | -0.42 |
| **DMQR Coping** | **Block 1** | 2.84* | .01 |  |  |
|  | **Gender** |  |  | .08 | 1.64 |
|  | **Age** |  |  | .09 | 1.89 |
|  | **Block 2** | 3.48 | .009 |  |  |
|  | **Gender** |  |  | .12 | 2.23 |
|  | **Age** |  |  | .09 | 1.89 |
|  | **EQ Affective** |  |  | -0.1 | -1.86 |
| **DMQR Enhancing** | **Block 1** | 1.3 | .007 |  |  |
|  | **Gender** |  |  | -.04 | -0.87 |
|  | **Age** |  |  | -.07 | -1.44 |
|  | **Block 2** | 0.18 | 0 |  |  |
|  | **Gender** |  |  | -.03 | -0.65 |
|  | **Age** |  |  | -.07 | -.1.44 |
|  | **EQ Affective** |  |  | -.02 | -0.42 |
| **DMQR Conforming** | **Block 1** | 0.08 | 0 |  |  |
|  | **Gender** |  |  | -.004 | -0.06 |
|  | **Age** |  |  | -.02 | -0.39 |
|  | **Block 2** | 2.22 | .006 |  |  |
|  | **Gender** |  |  | .02 | 0.49 |
|  | **Age** |  |  | -.02 | -0.04 |
|  | **EQ affective** |  |  | -.08 | -1.49 |
| **DMQR Socializing** | **Block 1** | 6.76*** | .03 |  |  |
|  | **Gender** |  |  | -.7 | -1.55 |
|  | **Age** |  |  | -.17 | -3.48*** |
|  | **Block 2** | 0.16 | 0 |  |  |
|  | **Gender** |  |  | -.08 | -1.59 |
|  | **Age** |  |  | -.17 | -3.43*** |
|  | **EQ Cognitive** |  |  | .02 | 0.4 |
| **DMQR Coping** | **Block 1** | 2.84* | .15 |  |  |
|  | **Gender** |  |  | .08 | 1.64 |
|  | **Age** |  |  | .09 | 1.89 |
|  | **Block 2** | 0.63 | .002 |  |  |
|  | **Gender** |  |  | .09 | 1.77 |
|  | **Age** |  |  | .09 | 1.81 |
|  | **EQ Cognitive** |  |  | -.04 | -1.79 |
| **DMQR Enhancing** | **Block 1** | 1.3 | .007 |  |  |
|  | **Gender** |  |  | -.04 | -0.87 |
|  | **Age** |  |  | -.07 | -1.44 |
|  | **Block 2** | 0.89 | .05 |  |  |
|  | **Gender** |  |  | -.06 | -1.14 |
|  | **Age** |  |  | -.06 | -.1.32 |
|  | **EQ Cognitive** |  |  | .07 | 1.37 |
| **DMQR Conforming** | **Block 1** | 0.08 | 0 |  |  |
|  | **Gender** |  |  | -.004 | -0.06 |
|  | **Age** |  |  | -.02 | -0.39 |
|  | **Block 2** | 0.06 | .002 |  |  |
|  | **Gender** |  |  | .05 | 0.1 |
|  | **Age** |  |  | -.02 | -0.46 |
|  | **EQ Cognitive** |  |  | -.04 | -0.77 |
| **DMQR Socializing** | **Block 1** | 6.76*** | .03 |  |  |
|  | **Gender** |  |  | -0.7 | -1.55 |
|  | **Age** |  |  | -.17 | -3.48*** |
|  | **Block 2** | 0.004 | 0 |  |  |
|  | **Gender** |  |  | -.07 | -1.49 |
|  | **Age** |  |  | -.17 | -3.47*** |
|  | **High pain scenarios** |  |  | -.003 | -0.06 |
| **DMQR Coping** | **Block 1** | 2.84* | .01 |  |  |
|  | **Gender** |  |  | .08 | 1.64 |
|  | **Age** |  |  | .09 | 1.89 |
|  | **Block 2** | 5.31* | .01 |  |  |
|  | **Gender** |  |  | .11 | 2.13 |
|  | **Age** |  |  | .09 | 1.84 |
|  | **High pain scenarios** |  |  | -.12 | -2.3* |
| **DMQR Enhancing** | **Block 1** | 1.3 | .007 |  |  |
|  | **Gender** |  |  | -.04 | -0.87 |
|  | **Age** |  |  | -.07 | -1.44 |
|  | **Block 2** | 2.31 | .006 |  |  |
|  | **Gender** |  |  | -.02 | -0.5 |
|  | **Age** |  |  | -.07 | -.1.48 |
|  | **High pain scenarios** |  |  | .08 | 1.52 |
| **DMQR Conformity** | **Block 1** | 0.08 | 0 |  |  |
|  | **Gender** |  |  | -.004 | -0.06 |
|  | **Age** |  |  | -.02 | -0.39 |
|  | **Block 2** | 4.85* | .01 |  |  |
|  | **Gender** |  |  | .02 | 0.43 |
|  | **Age** |  |  | -.02 | -0.45 |
|  | **High pain scenarios** |  |  | -.11 | -2.2* |
| **DMQR Socializing** | **Block 1** | 6.76*** | .03 |  |  |
|  | **Gender** |  |  | -0.7 | -1.55 |
|  | **Age** |  |  | -.17 | -3.48*** |
|  | **Block 2** | 3.7 | .009 |  |  |
|  | **Gender** |  |  | -.07 | -1.39 |
|  | **Age** |  |  | -.17 | -3.41*** |
|  | **Low pain scenarios** |  |  | -.09 | -1.92 |
| **DMQR Coping** | **Block 1** | 2.84* | .01 |  |  |
|  | **Gender** |  |  | .08 | 1.64 |
|  | **Age** |  |  | .09 | 1.89 |
|  | **Block 2** | 2.65 | .07 |  |  |
|  | **Gender** |  |  | .09 | 1.77 |
|  | **Age** |  |  | .1 | 1.96 |
|  | **Low pain scenarios** |  |  | -.08 | -1.62 |
| **DMQR Enhancing** | **Block 1** | 1.3 | .007 |  |  |
|  | **Gender** |  |  | -.04 | -0.87 |
|  | **Age** |  |  | -.07 | -1.44 |
|  | **Block 2** | 1.55 | .004 |  |  |
|  | **Gender** |  |  | -.04 | -0.77 |
|  | **Age** |  |  | -.07 | -.1.39 |
|  | **Low pain scenarios** |  |  | -.06 | -1.24 |
| **DMQR Conformity** | **Block 1** | 0.08 | 0 |  |  |
|  | **Gender** |  |  | -.004 | -0.06 |
|  | **Age** |  |  | -.02 | -0.39 |
|  | **Block 2** | 0.03 | 0 |  |  |
|  | **Gender** |  |  | .003 | 0.05 |
|  | **Age** |  |  | -.02 | -0.39 |
|  | **Low pain scenarios** |  |  | -.09 | -0.17 |

Note. *p<0.05 **p<0.01 ***p<0.001. N=387.
